# An outbreak of cutaneous anthrax in Yunnan, China

**DOI:** 10.1038/emi.2016.65

**Published:** 2016-06-22

**Authors:** Ying Huang, Yingrong Du, Yaling Wang, Ning Wang, Jinsong Bai, Haiyun Chen, Hua He, Jun Xu, Yan Wu, Yun Luo, Xiaolong Li, Guodong Liang

**Affiliations:** 1 The Third Hospital of Kunming City, Kunming 650041, China; 2 State Key Laboratory of Infectious Disease Prevention and Control, National Institute for Viral Disease Control and Prevention, Chinese Center for Disease Control and Prevention, Beijing 102206, China; 3 Collaborative Innovation Center for Diagnosis and Treatment of Infectious Diseases, Hangzhou 310058, China

**Dear Editor,**

Anthrax is an acute zoonotic infectious disease that is caused by *Bacillus anthracis*. Humans and other animals are susceptible to anthrax. Cattle, horses, sheep, camels and other herbivores are the main sources of infection in human anthrax cases. Anthrax infection in humans can occur through contact with sick animals or their products.^1^ Patients with cutaneous anthrax may present with skin necrosis, ulceration, extensive edema in the surrounding tissues, toxemia and other symptoms. Other forms of the disease include pulmonary anthrax caused by inhalation and intestinal anthrax caused by ingestion.^2, 3^ Because anthrax is very contagious, has severe manifestations, and can have an abrupt onset, every country classifies anthrax as a highly infectious disease.^1, 4^ Moreover, since the ‘anthrax powder' terrorist event that occurred in the United States in 2001, there has been a global concern regarding anthrax bioterrorism.^5^ This report describes an outbreak of anthrax in humans that occurred in Yunnan province, China, in 2015 following the slaughter of cows.

On October 30, 2015, the outpatient department of infectious diseases in the third hospital of Kunming (hereafter referred to as the hospital), received two cases (cases 1 and 2 in Table 1) in the morning and two more cases (cases 3 and 4 in Table 1) later that day. Blisters of different sizes were observed on both the hands and arms of each patient. All four patients were from the same village in Dongchuan, Kunming, Yunnan Province, China (25°57′ north latitude, 102°47′ east longitude). Dongchuan is located 150 km from Kunming.

By questioning the patients, the doctor learned that the four patients worked at a beef restaurant located in Dongchuan. The owner of the restaurant, who was one of the four patients, had purchased a head of sick cattle that was unable to walk from a local farmer at a cheap price, and it was immediately slaughtered at the restaurant. The four patients had aided in slaughtering the cattle on October 19, 2015. The slaughter commenced in the morning and was completed at noon on the same day. The beef and viscera were cooked for use in meals at the restaurant (the local villagers habitually eat beef). From 21 October, which was the third day following the slaughter, until 28 October, blisters appeared on the hands or arms of the four restaurant employees who had participated in the slaughter. Because no significant fever or other discomfort was noted, the affected individuals' blisters were dressed at a local clinic, and no other treatment was administered. As time passed, the number of blisters on the patients' arms increased, and all four patients experienced increasing pain. Therefore, a local doctor recommended that the patients seek treatment at the hospital in Kunming.

A body surface examination of each patient revealed diffuse blisters of various sizes (with diameters ranging from 0.5 cm to 5 cm) on the left or right hands, back or arms. Blister-like skin lesions were noted on the entire right arm in the fourth case. A yellowish exudate was observed in the lesions, the tissues surrounding the blisters were swollen, and some of the blisters resembled creamy white pustules, with swelling of the corresponding arms. The findings from our examinations and the degree of skin damage to the arms in the four cases are shown in Table 1. Because the four cases had a common history of exposure to cattle (perhaps infected cattle) and exhibited signs of anthrax (for example, vesicular skin lesions), the attending physician determined that the patients could have been infected with *B. anthracis* during the slaughter of the cow at the patients' place of employment. To avoid further spread of the disease, the hospital immediately transferred the four patients from the outpatient ward to the isolation ward, which is located in a different building. The isolation ward was designed to accommodate patients with major infectious diseases (for example, severe acute respiratory syndrome and human avian influenza), so the four patients in this ward were kept isolated from each other. The doctors and nurses maintained proper physical protection against infection during the diagnosis and treatment of the four cases. The medical staff bandaged the damaged skin of the patients to avoid further exposure to infection due to exudation. Routine blood tests revealed that neutrophils accounted for more than 85% of the white blood cell count in three of the four patients (Table 1). The hospital immediately reported the disease to the local center for disease control and prevention (CDC). The CDC collected samples of the exudate from the damaged skin of the patients in the hospital and carried out bacterial smears and cultures. On 31 October, the hospital received the CDC's report, which indicated that the bacterial smears and cultures for two of the four patients were positive for *B. anthracis*. On the basis of the patients' epidemiological information, clinical manifestations and positive anthrax test results, the doctors determined that the four patients had cutaneous anthrax due to infection with *B. anthracis* that was transmitted during the slaughter of ailing cattle. Although the patients were hospitalized, they were stable, and no pulmonary infection, nausea, vomiting or other complications occurred. After treatment, the edema in the patients' arms where skin had been damaged gradually subsided. In addition, diminished exudation was noted from the original blisters, and black scabs formed. On 17 November, the local CDC performed anthrax smears and bacterial cultures using new liquid samples collected from the four patients' damaged skin, and all of the results were negative. The four patients were discharged from the hospital on 20 November, 2015.

Anthrax is a natural focal disease with global distribution. Anthrax is most commonly found in pastoral areas of South America, Asia and Africa.^6^ Anthrax is a notifiable infectious disease in China, and each case must be promptly reported to health authorities.^7^ A total of 1911 cases of cutaneous anthrax were reported in 225 counties within 18 provinces from 2007 to 2011. Of these cases, 13 were fatal.^8^ Most of the cases were middle-aged individuals. Herders and farmers accounted for 86.08% of the total cases. In recent years, the number of anthrax cases has decreased in China.^9^ Anthrax cases in China are concentrated mainly in pastoral areas with poor economic and health conditions. Local herders are often unaware of how anthrax is transmitted and of the dangers of the disease; therefore, they are reluctant to discard dead animals and may skin them on-site for meat, resulting in infection. Cattle farming in Yunnan is common, and a large number of cattle is bred there. In addition, Yunnan is a natural focus of anthrax and several anthrax outbreaks related to the slaughter of cattle have been reported in recent years.^9^ The four reported cases in this study acquired anthrax during the slaughter of cattle. Because of timely treatment, the four patients fully recovered in a short time without complications. All of the patients returned home after treatment. In addition, the attending doctors quickly isolated the affected patients from the other patients at the hospital, thereby preventing the occurrence of secondary cases and further spread of the epidemic. As mentioned above, anthrax infections have occurred in some places in China. Until now, anthrax has been reported in mountainous areas, where cattle, sheep and other livestock are kept.^10^ Improving the management of sick animals and enabling herders to handle sick animals correctly by providing them with proper educational materials will help significantly reduce the number of human cases of anthrax due to contact with infected animals.

## Figures and Tables

**Table 1:** Clinical manifestations and laboratory test results for four cutaneous anthrax cases

**Number**	**Sex**	**Age (years)**	**Incubation (days)**	**Clinical manifestation**^a^	**Ratio (leukocyte:neutrophil)**^b^		**Etiological detection**^c^ **(admission/discharged)**
					**Admission**	**Discharged**	
1	M	53	8	Soybean-sized blisters on the right forearm	17.6:15.5	4.2:4.0	+/−
2	F	45	9	Blisters on the back of the left hand	13.5:11.7	6.5:3.8	+/−
3	M	45	2	Blisters on the hands	23.9:14.6	5.2:2.6	−/−
4	M	35	5	Blisters and severe edema on the right arm	13.7:11.6	4.5:1.1	−/−

a Clinical manifestations of patients admitted to the third hospital of Kunming on October 30, 2015.

b Blood tests (including leukocyte and neutrophil counts) were conducted using blood collected from the patients when they were admitted to the hospital and when they were discharged from the hospital.

c Smear cultures for *B. anthracis* were performed using fluid collected from lesions on the patients' arms before they were admitted to the hospital and after they were discharged from the hospital.
